# Lipids in Neurodegenerative Diseases

**DOI:** 10.3390/ijms241411523

**Published:** 2023-07-16

**Authors:** Valerio Chiurchiù

**Affiliations:** 1 Institute of Translational Pharmacology, National Research Council, 00133 Rome, Italy; valerio.chiurchiu@ift.cnr.it or v.chiurchiu@hsantalucia.it; Tel.: +39-06501703210; 2 Laboratory of Resolution of Neuroinflammation, IRCCS Santa Lucia Foundation, 00179 Rome, Italy

Lipids are undoubtedly the major constituents of the cell membranes of all living organisms, and the most efficient source of energy [1]. However, they also act as intercellular and intracellular signaling mediators by exerting many cell functions upon binding to specific G protein-coupled receptors and by interacting with them, thus modulating their localization and activation. Their ability to act as signaling mediators has earned them the name “bioactive lipids” [2]; said bioactive lipids are divided into four main families: classical eicosanoids, glycerophospholipids and sphingolipids, specialized pro-resolving lipid mediators, and endocannabinoids [3,4]. Although many neurodegenerative diseases are characterized by abnormal protein aggregates, with each pathologic aggregate having a distinctive temporo-spatial pattern of spread throughout the nervous system that is characteristic of a specific disorder, increasing evidence suggests that lipid membrane composition and the alteration of lipid metabolism play a major role in the propagation of neurodegeneration-associated protein aggregates throughout the brain [5].

In fact, we hinted at the multifaceted role that lipids have in pathophysiological cascades, which then lead to the most common (and, indeed, to the less common) neurodegenerative diseases, in the Editorial preceding this Special Issue “Lipids in Neurodegenerative diseases”, within the International Journal of Molecular Sciences. This valuable contribution to the field is made up of eight papers: five original articles and three reviews, providing new information about several classes of lipids under both normal and pathological conditions. 

In their review, Mandik and Vos [6] argue that despite it being well established that many neurodegenerative diseases are characterized by protein deposits, sphingolipids also take center stage in their underlying pathogenesis. The authors particularly focused on Parkinson’s disease (PD) and neurodegeneration with brain iron accumulation (NBIA), all characterized by extrapyramidal symptoms coupled to cognitive impairment and psychiatric disturbance [7]. The accumulation of subclasses of sphingolipids or of their byproducts (as well as defects in their metabolism) is typical of PD and NBIA with genetic mutations. The authors suggest not only that sphingolipid metabolism may be an interesting therapeutic target for these diseases, but also that a diet controlling the specific affected sphingolipids might provide a beneficial effect to patients.

Among the classes of sphingolipids, glycosphingolipids, which are all derived from lactosylceramide (LacCer) and include gangliosides, play key pathophysiological roles in vastly different processes including development, neuronal differentiation, and modulating receptor signaling [8].

Accordingly, Dei Cas et al. [9] developed a very accurate and sensitive novel approach to measure LacCer synthase activity in vitro using deuterated glucosylceramide via liquid chromatography coupled with tandem mass spectrometry; this method has the advantage of avoiding the costs and discomforts of managing radiochemicals. On the other hand, one of the main gangliosides, GM1, which is particularly expressed in the central nervous system, was shown to interact and form a stable complex with GPR37 in the article by Hertz et al. [10]. The authors showed the GPR37-dependent rescue effect of GM1 against MPP+, a common dopaminergic neurotoxin. This effect was observed in both stably and transiently overexpressing N2a neuronal cells, suggesting a direct effect of GM1 on the GPR37 signaling pathway, due to a reduction in the constitutive activity of GPR37 upon treatment with this ganglioside. Although these findings require further evaluation in vivo to elucidate their pathophysiological relevance, such an interaction is particularly important in light of the recent literature on one of the endogenous ligands for GPR37, namely the specialized pro-resolving lipid mediator (neuro)protectin D1, which exerts neuroprotective effects and is impaired in several neurodegenerative diseases [11,12,13].

The lipid composition of cell membranes is also important for the conformation and activity of basically all receptors; this was investigated in the study of Bershatsky et al. [14], which examined the insulin receptor subfamily that when impaired is associated with diabetes, cancer, and several neurodegenerative diseases. In this study, the authors suggest that the receptor activation and functioning within this subfamily may be membrane-mediated; this is due to the flexibility of their transmembrane domains that allows them to adapt to different lipid bilayers. Changes in fatty acids, specifically in palmitic acid (PA), were found in patients affected by the neurogenetic disorder named hereditary transthyretin amyloidosis, in the study by Luigetti et al. [15]; metabolic remodeling could be the basis of the mitochondrial dysfunction and neuroinflammation that are hallmarks of this disease.

Besides phospholipids, the main other lipid that makes up cell membranes is cholesterol; it plays key roles in regulating membrane fluidity and substrate presentation, alongside forming lipid rafts to assist in cell signaling and serving as a precursor for several biochemical pathways in brain cells [16]. One of the master regulators of plasma cholesterol levels is proprotein convertase subtilisin/kexin type 9 (PCSK9), and the study by Papotti et al. [17] showed that PCSK9 reduced the cholesterol supply to neurons from astrocytes by reducing apoE-HDL-mediated cholesterol uptake, and LDL receptor and apoE receptor 2 expression. PCSK9 also increased Aβ-induced neurotoxicity, suggesting that cholesterol deprivation has deleterious consequences for neuronal function and survival, making this enzyme an attractive pharmacological target for Alzheimer’s disease.

An overview of the relationship between lipoprotein metabolism and protein aggregation in Alzheimer’s disease, particularly in the so-called stress granules (SG) (non-membranous organelles that form in response to stress stimuli), is outlined in the review by Grao-Cruces [18]. These SG form during cellular stress and following the aggregation of misfolded proteins, and are likely to protect against cell death.

Lastly, our contribution reviewed all the methodological approaches performed to detect and quantify the different classes of lipids in the brain tissues, cerebrospinal fluid, or serum/plasma of patients affected by Alzheimer’s and Parkinson’s disease, and reviewed their respective animal models [19]. We have concluded that in order to gain a comprehensive understanding of these lipids’ roles in the pathogenesis of these disorders, not only are multi-omic studies required to analyze simultaneously all these lipids and the other macromolecules (i.e., proteins, carbohydrates, and nucleic acids) with which they interact; standardized techniques are warranted to minimize interlaboratory differences, and to advocate for their potential use as biomarkers of diagnosis, progression, activity, and response to different therapies. Bioinformatical tools could also be of help in predicting cell metabolism and dynamics in order to facilitate experimental validation both in vitro and in vivo [20].

Interestingly, all the studies focused on restricted classes of lipids, and no papers proposed for this Special Issue primarily addressed the manifold bioactive lipids that signal both paracrinally and autocrinally by regulating key neuroinflammatory processes, which currently seem to represent one of the most regularly studied features that the different neurodegenerative diseases have in common.
